# Platform for Drug Testing and Studying Rapid-Onset Signaling and Induction of Cellular Phenotypes *Ex Vivo*

**DOI:** 10.1016/j.jid.2025.05.033

**Published:** 2025-06-12

**Authors:** Helene Dworak, Karla Valdivieso, Barbara Schädl, Nadja Anneliese Ruth Ring, Tomaz Rozmaric, Paul Slezak, Johannes Grillari, Heinz Redl, Mikolaj Ogrodnik

**Affiliations:** 1 Ludwig Boltzmann Institute for Traumatology. The Research Center in cooperation with AUVA, Vienna, Austria; 2 Austrian Cluster for Tissue Regeneration, Vienna, Austria; 3 University Clinic of Dentistry, Medical University of Vienna, Vienna, Austria; 4 Institute of Molecular Biotechnology, BOKU University, Vienna, Austria

## Abstract

The development of drugs and the assessment of their efficacy require reliable platforms that can mimic *in vivo* conditions. Our research has demonstrated that *ex vivo* skin exhibits numerous core phenotypes following injury that serve as subjects for research and targets for drug development, including cellular senescence, apoptosis, inflammation, mTOR and Erk signaling pathways, among others. In this context, we present a protocol for establishing an *ex vivo* skin platform, including skin preparation, biopsy collection, targeted drug application, tissue incubation, fixation, and the evaluation of drug effects on signaling pathways and cellular phenotypes. Although fundamentally simple in nature, our protocol is designed to be accessible to any laboratory interested in studying fundamental tissue damage responses and the mobilization of cells for repair. We believe this protocol will serve as a cost-effective, reliable, and reproducible tool consistent with the 3Rs methodology for research, clinical applications, and industry use.

## To The Editor

Research on molecular signaling and the induction of cellular phenotypes in live tissues faces numerous challenges. For one, conducting experiments in rodents necessitates sacrificing animals to collect samples, which limits the availability of sufficient time points, conditions, sexes, age groups, and so on. Additionally, almost without exception, each animal yields only one sample per organ of interest. Furthermore, rodent tissues differ significantly from human tissues, particularly skin^1^. While *in vitro* models can offer robust alternatives, systems such as organ equivalents and organoids often fail to accurately replicate native human tissues^2^.

Research of recent years shows that tissue physiology operates not only on scale of days-weeks, but that rapid processes also occur minutes- hours after a stimulus^3^. We, and others have shown that activation of signaling pathways such as Erk and mTOR or cellular phenotypes such as cellular senescence, can occur rapidly, within the first minutes (signaling pathways activation) or hours (cellular phenotypes) upon stimulation *in vivo*^3–8^. These and other rapid signals^3,4^, were found to control injury-induced properties of cells such as migration, inflammation, proliferation and others in plants^5^, invertebrate^6^, fish^7^ and mammals^4,8^.

Porcine skin models have been extensively used for drug delivery studies because they offer similar barrier properties, composition, and thickness to human skin^9^. Despite the utility of porcine models, existing techniques often lack the means required to evaluate drug activity and have limited potential for studying the biology of skin damage responses.

To address these gaps, our research introduces a novel *ex vivo* platform and methodology for studying the rapid-onset of signaling and the induction of cellular phenotypes following drug application to injured skin. This platform allows for a detailed evaluation of cellular responses, including senescence, apoptosis, inflammation, and activation of key signaling pathways such as mTOR and Erk. Our approach has already been successfully applied in previous studies, where we used the platform to evaluate the induction of p-rpS6-zone^4^ and cellular senescence^8^.

**Figure 1:**
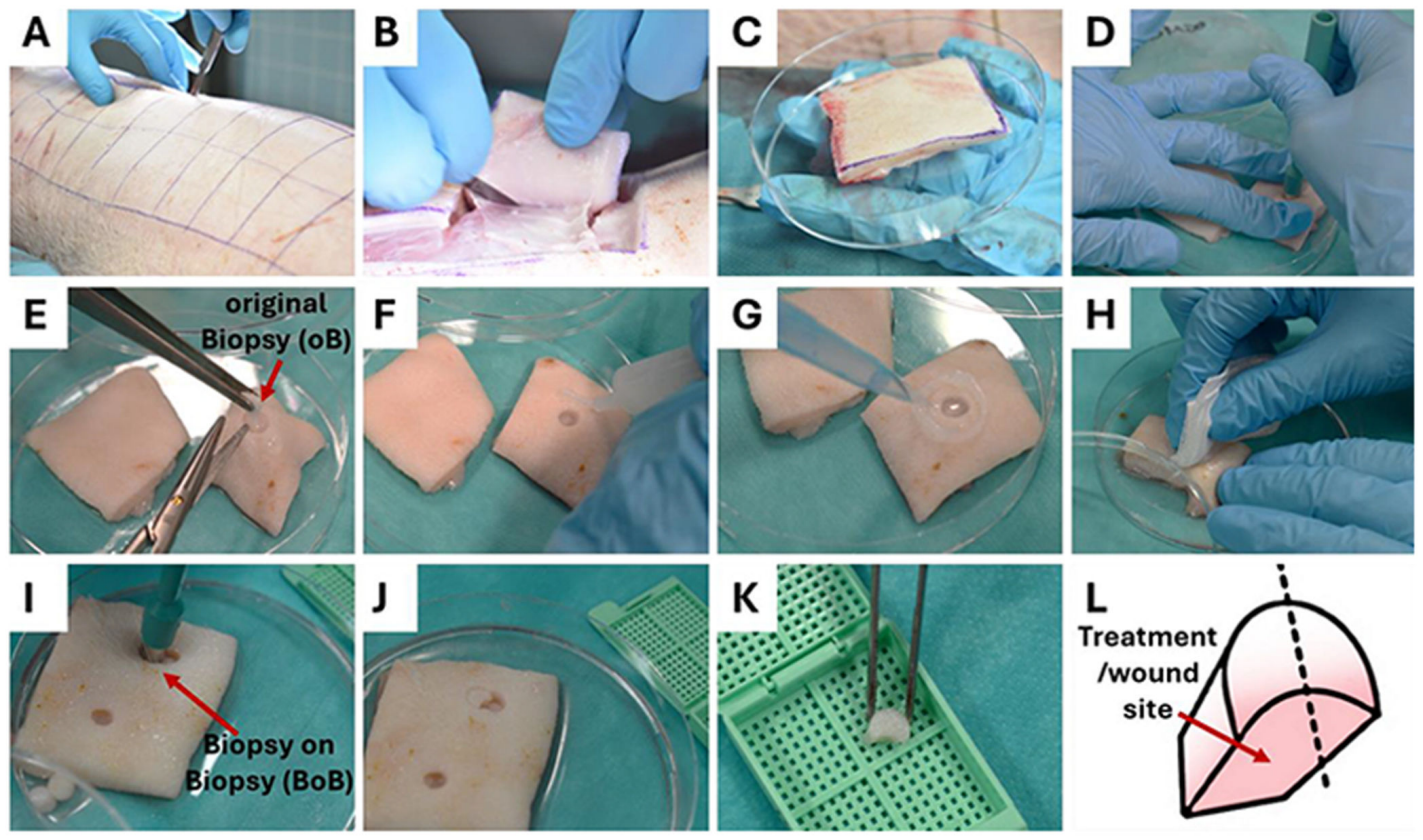
*Ex vivo* workflow for skin sample preparation and drug application. **A. Skin preparation:** Excise/harvest a skin sample (above muscle). **B. Skin cutting:** Cut the excised skin sample into uniform square pieces using a scalpel. **C. Incubation:** Incubate the skin in a humidified CO_2_ incubator at 37°C for 1 hour. **D. Biopsy punch:** Use a 6 mm biopsy punch to extract a uniform section of skin tissue. **E. Cutting off the biopsy:** Cutting of the biopsy on its base with scissors. **F. Preparation of hydrophobic barrier:** Apply a Vaseline ring around the biopsy wound to maintain a concentrated drug environment and prevent solution leakage. **G. Drug application:** Introduce the drug solution directly into the biopsy to ensure optimal soaking and penetration into the tissue. **H. Hydrophobic barrier cleaning:** Carefully clean the hydrophobic Vaseline barrier after drug application and incubation. **I. Secondary biopsy punch:** Following incubation, take a second biopsy (half-moon shape) from the edge of the treated area to study drug penetration. **J. Excision of the second biopsy punch:** Carefully excise this second biopsy (Biopsy on Biopsy; BoB) using scissors. **K. Fixation in histocassette:** Place the excised biopsy into a histocassette and immerse it in 10% Formalin for fixation, ensuring the sample is preserved for histological analysis. **L. Plane of sectioning:** Orientation of the tissue sectioning during the histological preparation.

In this Letter, we present a detailed, step by step protocol (Fig. 1; protocol in the Supplementary Materials and Methods) for the preparation, drug application and processing of skin samples in an *ex vivo* model. While the protocol described here is based on the use of porcine skin, the same methodology (for details see the Supplementary Materials and Methods) can be used for human *ex vivo* skin (collected as a waste product e.g. from lipoplasty).

At its core, the method is very simple, creating an excision injury cavity that can hold ~200 μl of solution. Following euthanasia of a pig, samples should be excised (Fig. 1A-C) immediately. However, in our experience, samples collected within 2 h are not affected. Our published results indicate that if samples are collected from human subjects, general anesthesia has no effect on the observed wounding responses^4^. To evaluate how drug modulates cellular responses, we employ a two-step biopsy process called “Biopsy on Biopsy” (BoB). This involves an initial biopsy (“original Biopsy”; oB) followed by drug application (Fig. 1D-H). A hydrophobic barrier (a Vaseline ring) is carefully placed around the biopsy wound to prevent leakage and maintain a concentrated drug environment, followed by the addition of the drug solution within the hydrophobic barrier (Fig. 1G). After incubation, the wound must be cleaned gently with cotton swabs to remove the drug solution and the Vaseline ring completely without damaging the tissue (Fig. 1H). Following the incubation period, a second biopsy (BoB) is taken at the edge of the initial biopsy (Fig. 1I-L). The collected sample is immediately fixed in formalin for 24 hours for subsequent analysis. Adherence to key considerations, including incubation timing, careful application of the hydrophobic barrier to prevent leakage, and gentle tissue handling to preserve its integrity, is essential for obtaining reliable results. When conducting the experiment, a minimum of four biological replicates (i.e., four separate pieces of skin or ideally four pigs per group) is recommended to account for inter-animal variability and provide sufficient statistical power for quantitative analyses. Potential problems and troubleshooting are presented in the Supplementary Figure 1 and Supplementary Table 1, respectively.

**Figure 2:**
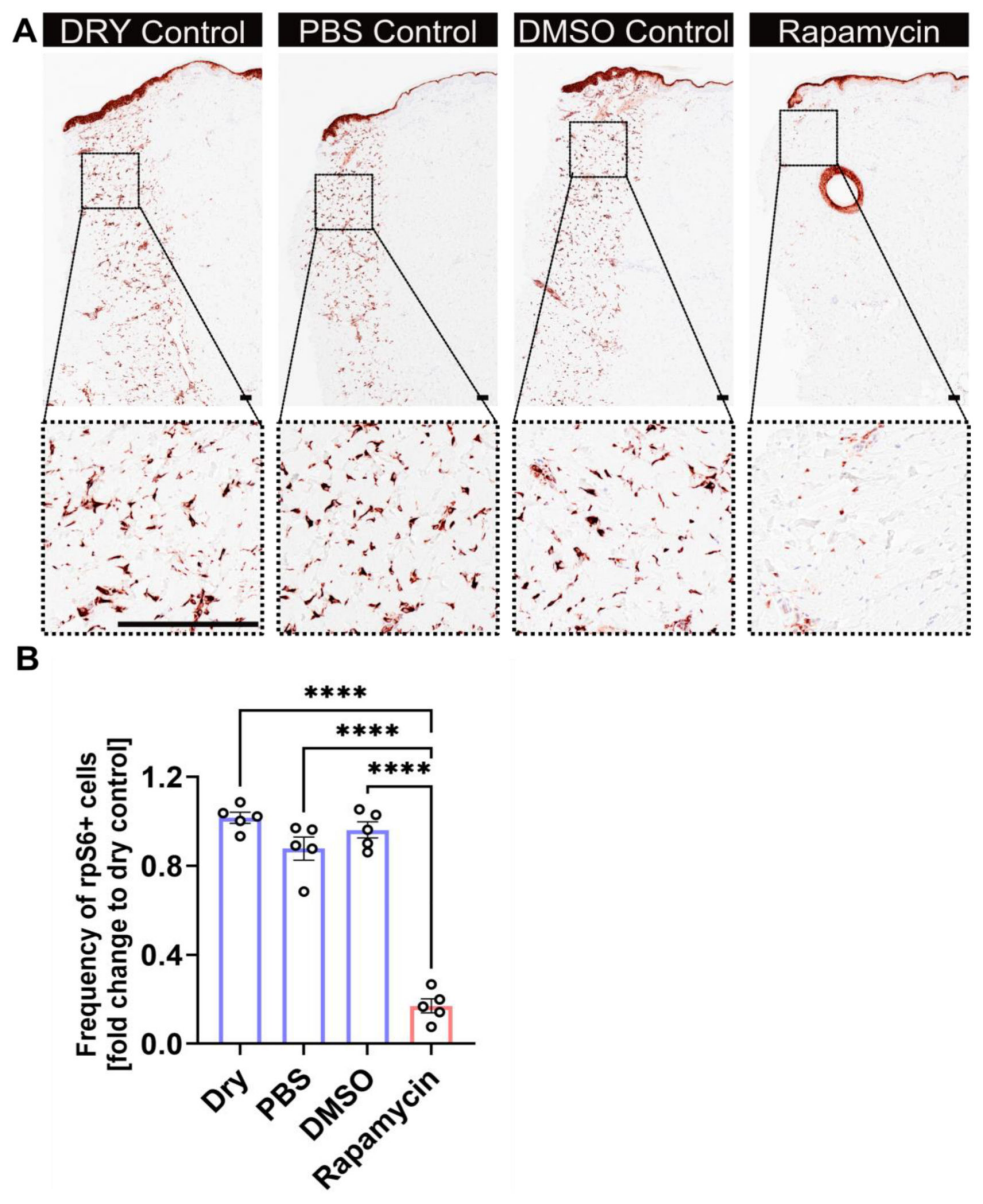
Evaluation of drug effects in the platform. **A**. Representative images of p-rpS6 in samples collected from different controls (DMSO, PBS, Dry) and from a positive control, mTOR inhibitor (Rapamycin; 55 μM in PBS). **B**. Particle analysis per histological section. Data are from n = 5 pigs per group. Mean ± SEM plotted. For (B) one-way ANOVA with Dunnet’s post hoc test was used. ****p<0.0001. The scale bars for all images are 100 μm.

To demonstrate the robustness and reliability of the platform, we implemented three distinct control conditions (Fig. 2A):

### Dry

Wounded, but otherwise untreated control condition of skin *ex vivo*, providing a reference for tissue behavior upon an injury without the influence of external solutions, serving as a baseline for comparison.

### PBS

A physiologically isotonic solution that is administered directly to the wound cavity and can be used as a solvent for hydrophilic drugs.

### DMSO

Widely used as a solvent for drug delivery, DMSO control helps to evaluate any potential confounding effects introduced by the carrier solution itself. We tested solvent concentrations of 0.1, 0.5 and 1% (in PBS), having no impact on the development of the wounding signals.

As a positive control drug to affect activation of wounding response, we use **Rapamycin**, an inhibitor targeting the mTOR pathway, resulted in a significant reduction in p-rpS6 induced by an injury (Fig. 2A-B). This reduction validates the platform’s sensitivity and its ability to detect specific signaling responses, highlighting its utility for precise evaluation of drug effects on cellular pathways.

In this Letter, we provide proof-of-concept evidence demonstrating the suitability of the platform for drug testing and modulation of rapid-onset damage signals in wounds. Specifically, we quantify p-rpS6 induction by injury in porcine skin *ex vivo* and show its flexibility in modulation using Rapamycin. We have chosen p-rpS6 as it is induced as an evolutionarily conserved mechanism of injury response across humans, mice, pigs^4,8^ and axolotls^10^. However, in our other works, we demonstrate the suitability of this approach for investigating additional rapid-onset events following injury, such as the induction of c-Fos, p-Erk, p21 (*CDKN1A*), DNA damage, proliferation markers, and the downregulation of HMGB1 and LMNB1^4,8^. The fast progression of research into rapid-onset tissue damage responses is likely to uncover more markers and applications suitable for this platform. In summary, this Letter introduces a protocol designed to be a cost-effective, reliable, and reproducible tool aligned with the 3Rs methodology, suitable for research, clinical applications, and industrial use in the context of tissue damage and repair.

## Supplementary Material

Supplementary Data

## Data Availability

The data generated and/or analyzed during this study are available from the corresponding author on reasonable request at: mikolaj.ogrodnik@lbg.ac.at No large datasets were generated or analyzed during this study
