# Supplementary material for: Platform for Drug Testing and Studying Rapid-Onset Signaling and Induction of Cellular Phenotypes Ex Vivo
Source: J Invest Dermatol. Author manuscript; Available in PMC 2026 Aug 3. (PMC7619302; doi:10.1016/j.jid.2025.05.033)
Supplement: Supplementary Data [file EMS216207-supplement-Supplementary_Data.docx]

**SUPPLEMENTARY MATERIALS AND METHODS**

**Reagents**

- 10% Formalin (4% formaldehyde) for fixation
- DMEM-F12 medium + 1% PenStrep (penicillin-streptomycin)
- Phosphate-buffered saline (PBS) + 1% PenStrep
- Drug solution (appropriate for the experiment). For the positive control of the Figure 2, 10 mg of Rapamycin was dissolved in 200 µL of DMSO to prepare a 55 mM stock solution. For drug administration to the tissue, the stock solution was diluted 1:1000 in PBS to a final concentration of 55 µM, and this solution was added to the wound cavity.
- Vaseline (for creating a hydrophobic barrier)

**Equipment**

- 6 mm biopsy punch
- Scalpel
- Scissors
- Tweezers
- Petri dish
- Timer
- 5mL syringe (filled with Vaseline)
- Styrofoam lid (as a temperature-insulating base)
- Cotton swabs
- Histocassettes (for tissue storage during fixation)
- Incubator (set to 37°C)
- Slides
- Liquid blocker pen
- Coverslips
- Light microscope and imaging software
- Software program that enables analysis (e.g., ImageJ/Fiji or QuPath)

**Procedure**

1. **Skin preparation**:

Euthanize the animal and excise a skin sample (5cm x 5cm) using a scalpel (Fig. 1A-B). Preferably, use skin from the side or back of the animal. From a 5 cm × 5 cm skin piece, two biopsies can be obtained from opposite sides of the tissue. We recommend maintaining a minimum distance of 1.5 cm between biopsies and avoiding proximity to the skin edges to prevent overlapping influences from wound edges. In other words, the distance between biopsies (or between a biopsy and the edge) should be at least 1.5 cm to ensure that wounding responses from one injury do not influence the others.

1. **Initial processing**:

Place the excised skin into a Petri dish (Fig. 1C).

Gently wash the skin piece 3 times with PBS + 1% PenStrep in a small beaker.

Add 2–3mL of DMEM-F12 medium + 1% PenStrep to the Petri dish.

For potential use of human skin samples, we recommend an additional antiseptic wipe with a sterile PBS + antibiotic/antimycotic solution before incubation to minimize contamination risk, while avoiding aggressive disinfectants that could damage the tissue.

1. **Incubation/acclimatization**:

Incubate the skin sample in a humidified CO₂ incubator at 37°C for at least 30 minutes up to 1 hour.

1. **Perform initial biopsy and add drugs**:

Use a 6 mm biopsy punch to take an original biopsy (oB) of the skin (Fig. 1D), but avoid punching through the subcutaneous tissue completely.

With scissors and tweezers, carefully cut through the epidermis and dermis until you reach the subcutaneous tissue (Fig. 1E) to generate a proper cavity.

Immediately fill the cavity with 200µL of drug solution.

1. **Creating a hydrophobic barrier**:

Create a hydrophobic barrier with a circle of Vaseline around the biopsy using a Vaseline-filled syringe (Fig. 1F). Ensure the Vaseline does not enter the wound.

Add another 200µL of drug solution within the Vaseline circle to ensure thorough soaking of the biopsy wound (Fig. 1G). The biopsy wound should be completely covered by the solution.

1. **Incubation**:

Close the Petri dish carefully and place it back in the incubator at 37°C.

Incubate for 1 hour. However, this duration can be shortened to 30 minutes if the user aims to study signaling pathways with less stability than those we described for p-rpS6, such as p-Erk induction. The protocol remains flexible in this regard, so we defer this adjustment to the user.

1. **Wound cleaning**:

After incubation, remove the dish from the incubator.

Wipe off the remaining liquid and Vaseline (Fig. 1H) carefully to avoid entering Vaseline into the wound.

Clean and dry the wound gently with a cotton swab without damaging the tissue to remove the excess of the drug.

1. **Final incubation**:

Place the skin sample in the incubator for an additional 30 minutes. The incubation duration ultimately depends on the specific biological process being studied. In our experience, 30 to 90 minutes is sufficient to investigate signaling pathways such as p-rpS6 and p-Erk, and certain cellular phenotypes, such as cellular senescence. We have successfully maintained satisfactory tissue structure and function in these assays for up to 24 hours, allowing for the study of a wide range of topics related to wound healing and tissue damage responses.

1. **Second biopsy (half-moon biopsy)**:

After a set time period, take another 6 mm biopsy at the edge of the initial biopsy site (Fig. 1I), creating a half-moon shape biopsy (BoB) (Fig. 1J-L).

1. **Fixation**:

Immediately place the tissue sample into a histocassette and immerse it in 10% Formalin for 24 hours. Wash the sample as needed and proceed with paraffin embedding and histological processing.

**SUPPLEMENTARY TABLES**

**Table 1. Troubleshooting**

| **Problem** | **Possible reason** | **Solution** |
| --- | --- | --- |
| 1. Drug solution leaking from biopsy site; resulting in only the lower part of tissue affected by drug (Fig. S1A) | Incomplete or poorly applied hydrophobic Vaseline barrier | Ensure Vaseline barrier fully encircles the biopsy site with no gaps; apply a second layer if necessary. Use a smaller syringe for more precise application. |
| 1. Area affected by wounding response is too thin or irregular, resulting in only the lower part of the edge being an actual wound (Fig. S1B) | Skewed or incomplete biopsy collection during the second biopsy | Make sure that the biopsy punch is held vertically and does not slide when collecting a biopsy; this potentially increases the area of collection for the second biopsy |

**SUPPLEMENTARY FIGURES**

**
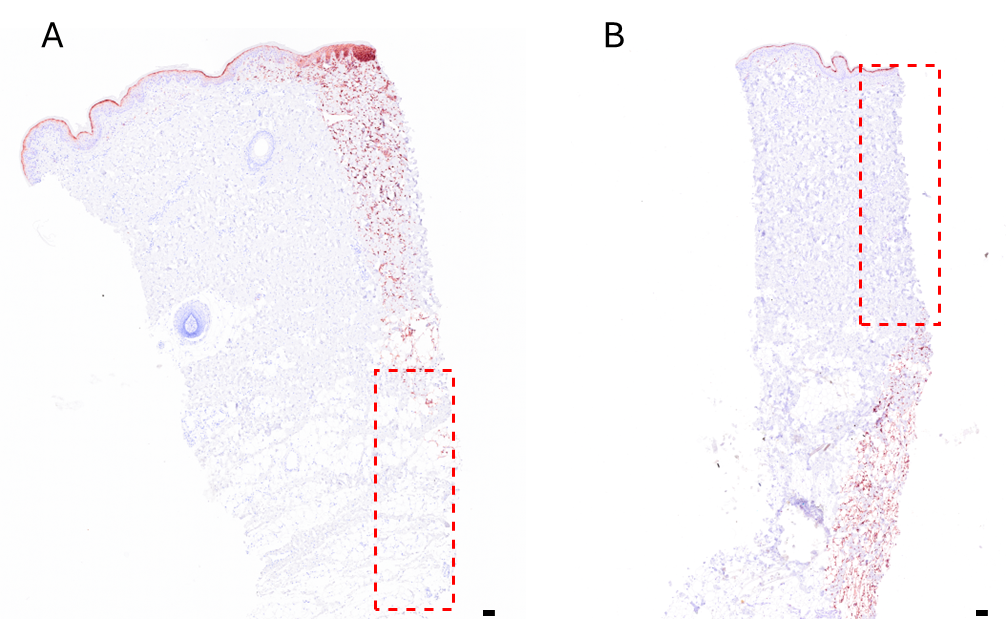
Supplementary Figure 1**

**Supplementary Figure 1. Factors impacting reproducibility in drug response and biopsy collection.**

Representative images of p-rpS6 in samples, illustrating factors affecting biopsy consistency and drug response.

**A.** Due to drug leakage, only the lower tissue layers are affected.

**B.** Irregular wound area due to incomplete biopsy collection.

Red frames mark regions where the lack of staining is caused by improper processing of the tissue samples. The scale bars for all images are 100 μm.
